# Calycosin-7-O-β-D-Glucoside Facilitates Axonal Regrowth and Functional Recovery via Rho/ROCK Pathway Inhibition After Cerebral Ischemia/Reperfusion

**DOI:** 10.3390/ijms27104469

**Published:** 2026-05-16

**Authors:** Pengcheng Wang, Aiming Yu, Yingxi Liang, Lisheng Wang

**Affiliations:** 1 Department of Pharmacology and Toxicology, School of Pharmaceutical Sciences, National and Local United Engineering Lab of Druggability and New Drugs Evaluation, Guangdong Provincial Key Laboratory of New Drug Design and Evaluation, Sun Yat-sen University, Guangzhou 510006, China; wangpch9@mail2.sysu.edu.cn; 2 College of Chinese Materia Medica, Guangzhou University of Chinese Medicine, Guangzhou 510006, China

**Keywords:** calycosin-7-*O*-β-D-glucoside, ischemia/reperfusion, Rho/ROCK pathway, axonal regeneration

## Abstract

Calycosin-7-O-β-D-glucoside (CG), a bioactive compound extracted from the traditional Chinese herb Astragalus (AR), exhibits diverse biological activities, including anti-oxidative and anti-inflammatory effects, and has shown protective properties in ischemia–reperfusion (I/R) injury. While previous studies have demonstrated that CG mitigates I/R injury primarily through its anti-oxidative and anti-inflammatory actions, its potential role in promoting neuroregeneration—a critical process for stroke recovery—remains unclear, and the underlying mechanisms have yet to be elucidated. In this study, an ischemic stroke model was established in rats via middle cerebral artery occlusion (MCAO). Seven days after CG treatment, cerebral infarct volume was assessed using triphenyltetrazolium chloride (TTC) staining, while neurological function was evaluated through behavioral tests. Nissl staining and Bielschowsky silver staining were employed to examine neuronal damage and axonal loss, and immunofluorescence was used to assess axonal regeneration. The expression of key proteins in the Rho/ROCK signaling pathway was analyzed by Western blotting (WB) and quantitative real-time PCR (qRT-PCR). CG treatment significantly reduced infarct volume, promoted axonal regeneration, improved neurological outcomes, and modulated the expression of RGMa, Rho, ROCK, and CRMP2. Collectively, these findings provide the first evidence that CG facilitates axonal regeneration and neurological recovery after cerebral ischemia, at least in part by inhibiting activation of the Rho/ROCK pathway, highlighting its potential as a therapeutic agent for ischemic stroke.

## 1. Introduction

Ischemic stroke (IS) is a rapidly developing brain disorder that accounts for the vast majority of strokes [1]. In the early stages of stroke, brain damage is often accompanied by impaired axonal atrophy resulting in neurotransmission defects. Therefore, stroke patients commonly have disabilities that involve severe language, cognitive, and physical dysfunctions [2]. Exercise rehabilitation training for IS patients or animal models can fix the nerve fibers that control muscle movements, suggesting that promoting prolongation of atrophic axons can significantly improve recovery of neurological function [3].

The Ras homolog gene /Rho-associated protein kinase (Rho/ROCK) pathway is involved in maintaining microtubule and cytoskeletal stability, which is important for growth cone formation during axonal regeneration [4]. Surface receptors of the growth cone can recognize various growth signals received in the neurons and guide the axon to extend in the correct direction. Repulsive guidance molecule a (RGMa) is a potent inhibitor of axonal regeneration that is expressed in the developing and mature central nervous system. After a stroke, upregulated RGMa binds to the specific surface receptor on the growth cone and activates the Rho/ROCK pathway, eventually destabilizing the cytoskeleton and causing the growth cone to collapse [5]. Inhibiting RGMa or treatment with ROCK inhibitors can stimulate recovery of motor function, which is possibly due to the promotion of axonal regeneration [6,7]. Therefore, the inhibition of axonal regeneration is dependent on activation of this signaling pathway.

As a widely used herb in traditional Chinese medicine, AR exhibits various bioactivities, such as anti-inflammatory, anti-oxidative, immunoregulatory and certain neuroregeneration effects [8,9,10]. CG is a flavonoid compound isolated from AR [11]. In addition to its pharmacological potential, the translational feasibility of CG should also be considered. As a naturally derived flavonoid compound from Astragalus Radix, CG may offer practical advantages for drug development, including a defined chemical identity and potential accessibility as a bioactive monomer. Moreover, Astragalus Radix and its related constituents have been widely used in traditional medicine, which provides a preliminary basis for considering the tolerability of CG. However, the safety profile, optimal dosing range, pharmacokinetic characteristics, and clinical feasibility of CG in the setting of ischemic stroke remain to be systematically evaluated. Our previous research demonstrated that CG can reduce oxidative stress and neuronal apoptosis [12], and improve expression of GAP43 in a PC12 cell oxygen-glucose deprivation/reperfusion (OGD/R) model, suggesting that GG can promote axonal regeneration [13]. Nevertheless, how CG exerts protection for I/R injury is unclear. This shows that CG is a promising candidate drug in stimulating neuroregeneration for I/R injury treatment.

In this study, we established an animal model of middle cerebral artery occlusion (MCAO) to investigate the effects and mechanism of CG on improving axonal regeneration and functional outcomes after I/R injury. We demonstrated that CG promoted axonal regeneration and neurological recovery after IS by inhibiting the Rho/ROCK pathway. Our study provides insight into the mechanism for promoting stroke rehabilitation and empirical evidence for its use in the clinic.

## 2. Results

### 2.1. CG Promotes Neurological Recovery After Ischemia–Reperfusion (I/R) Injury

**Figure 1:**
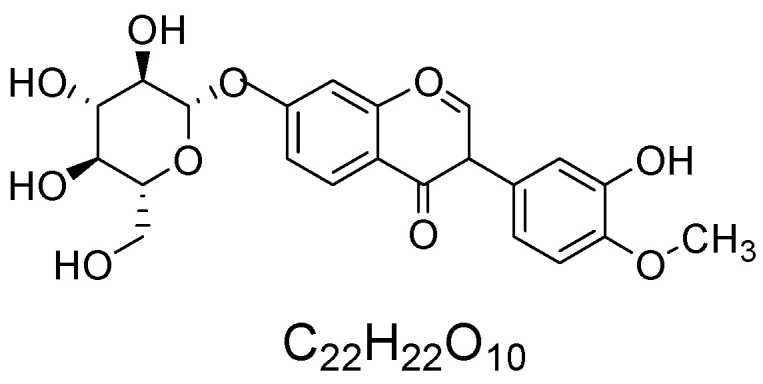
Structure and chemical formula of CG.

Calycosin-7-O-β-D-glucoside (CG) is a constituent originally isolated from Astragalus Radix (AR) (Figure 1). For the subsequent experiments in this study, a commercially available purified CG standard was used, and its purity was confirmed to be above 98% by high-performance liquid chromatography (HPLC) analysis.

**Figure 2:**
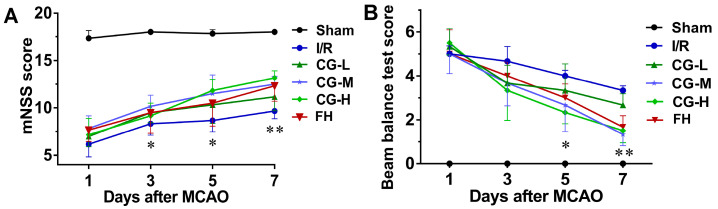
Effect of CG on neurological function. (**A**) Analysis of mNSS scores. (**B**) Analysis of BBT scores. Data are presented as mean ± SD (n = 5). * *p* < 0.05, ** *p* < 0.01 vs. I/R group.

The neurological function outcomes for the Sham, I/R, CG, and fasudil hydrochloride (FH) groups are shown in Figure 2A,B. On day 3, rats treated with CG, particularly those in the high-dose CG (CG-H) group, as well as the FH group, showed significantly improved mNSS scores compared with the I/R group (*p* < 0.05). In contrast, no significant differences in balance beam test (BBT) scores were observed between the I/R group and any of the CG-treated groups or the FH group at this time point. On days 5 and 7, rats in the middle-dose CG (CG-M), CG-H, and FH groups exhibited significantly improved mNSS scores compared with the I/R group (*p* < 0.01). These findings indicate that CG treatment alleviated neurological deficits and promoted functional recovery after ischemic stroke.

### 2.2. CG Reduces Cerebral Infarction and Edema After I/R Injury

**Figure 3:**
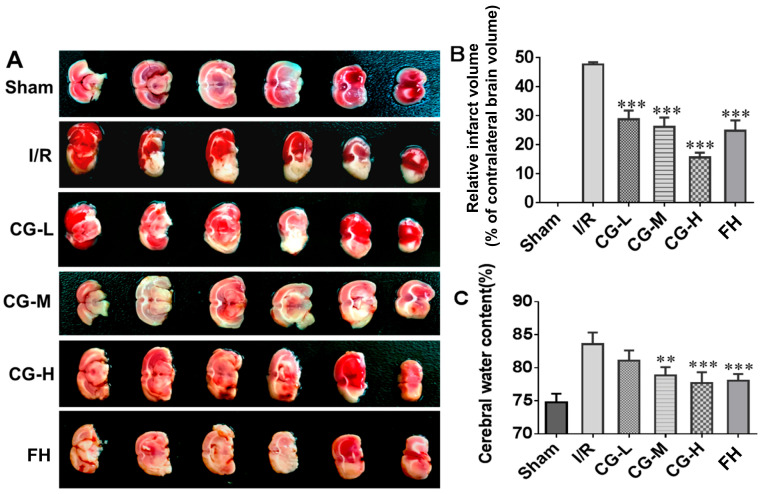
Images of cerebral infarction and cerebral edema after seven days of CG administration. (**A**) Image of TTC staining. (**B**) The relative size of infarct volume (% total volume of the contralateral brain) of each group. (**C**) Analysis of cerebral edema of each group. Data are presented as mean ± SD (n = 5). ** *p* < 0.01, *** *p* < 0.001 vs. I/R group.

TTC staining revealed no infarcted regions in the coronal brain sections of the Sham group, whereas a well-demarcated pale infarct area was observed in the I/R model group (Figure 3A). Administration of CG at different doses and FH significantly reduced cerebral infarction compared with the untreated I/R group (** *p* < 0.001, Figure 3B). Consistently, brain edema was markedly alleviated following 7 days of CG or FH treatment. Among the CG-treated groups, the high-dose CG-H group exhibited the most pronounced neuroprotective effect, with the lowest infarct rate (15.63 ± 1.63%) and cerebral water content (77.69 ± 1.65%). These findings indicate that CG effectively protects against cerebral ischemic injury following stroke.

### 2.3. CG Attenuates Histology and Neuron Damage After Neurological Impairment

**Figure 4:**
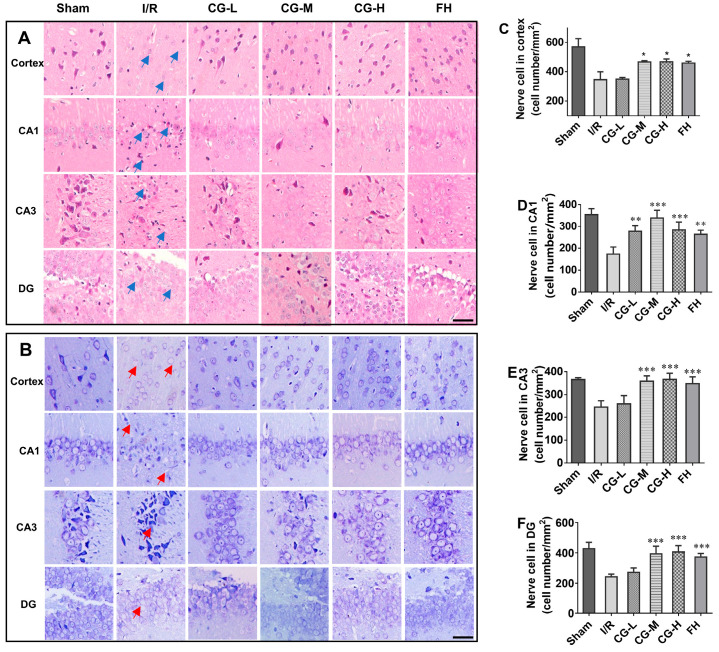
Images of cerebral pathology after HE and Nissl staining showing histological and neuronal damage in rats sacrificed at day 7 after I/R injury. (**A**) HE staining images. Scale bar = 50 μm. (**B**) Nissl staining images. Scale bar = 50 μm. (**C**) Quantitative analysis of cortical neurons. (**D**) Quantitative analysis of hippocampus CA1 neurons. (**E**) Quantitative analysis of hippocampus CA3 neurons. (**F**) Quantitative analysis of hippocampus DG neurons. Data are presented as mean ± SD (n = 5). * *p* < 0.05, ** *p* < 0.01, *** *p* < 0.001 vs. I/R group.

Given the beneficial effects of CG on post-stroke recovery, histopathological changes were next assessed using HE and Nissl staining. HE-stained images (Figure 4A) revealed that, in the Sham group, the cortical and hippocampal structures were intact and clearly defined, with densely arranged neurons and well-organized cellular layers. Neuronal nuclei were round or oval and basophilic, the cytoplasm was abundant and lightly stained, the nuclear-to-cytoplasmic boundaries were clear, and no obvious interstitial edema was observed. The hippocampal subregions were also clearly distinguishable. In contrast, the I/R group exhibited marked pathological injury in both the cortex and hippocampus, including loose tissue arrangement (blue arrows), neuronal shrinkage, nuclear pyknosis, hyperchromasia, fragmentation, eosinophilic cytoplasmic condensation, and microvacuole formation. The intercellular spaces surrounding neurons were enlarged. In addition, cortical pyramidal neurons showed obvious degeneration and necrosis, with a marked reduction in neuronal number. In the hippocampus, neurons were disordered, cell boundaries were indistinct, and the layered structure was severely disrupted, particularly in the CA1 region. Compared with the I/R group, rats treated with low-, medium-, and high-dose CG showed varying degrees of improvement in neuronal morphology, cellular arrangement, and tissue architecture, along with reduced neuronal pyknosis and necrosis.

Nissl staining, which highlights basophilic Nissl bodies in neuronal cytoplasm, was used to evaluate neuronal integrity. As shown in Figure 4B, neurons in the Sham group had normal morphology, with clear round or oval nuclei and abundant, densely distributed Nissl bodies in the cytoplasm, and no obvious interstitial edema. In the I/R group, extensive neuronal degeneration and necrosis were observed in both the cortex and hippocampus, accompanied by disorganized arrangement, shrinkage of the cell body and nucleus, and dissolution of Nissl bodies (red arrows). In contrast, the low-dose CG (CG-L), CG-M, CG-H, and FH groups showed clearer Nissl bodies and less severe neuronal damage than the I/R group. Quantitative analysis further demonstrated that the numbers of surviving neurons in the cortex and hippocampus were significantly higher in the CG-treated groups than in the I/R group (Figure 4C–F).

### 2.4. CG Alleviates Axonal Degeneration After I/R Injury

**Figure 5:**
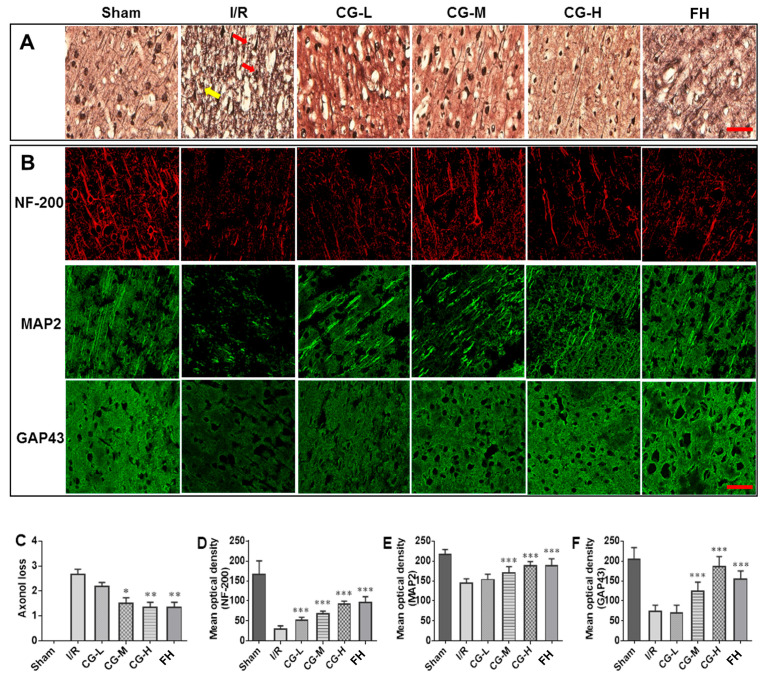
BSSM staining and IF staining in tissues from rats sacrificed on day 7 after I/R injury. (**A**) Images of neurons after BSSM staining. Scale bar = 50 μm. (**B**) Representative IF images showing NF-200, MAP2, and GAP43 localization and expression. Scale bar = 20 μm. (**C**) Semi-quantitative analysis of axonal loss. (**D**–**F**) Semi-quantitative analysis of fluorescence intensity of NF-200, MAP2 and GAP43. Data are presented as mean ± SD (n = 5). * *p* < 0.05, ** *p* < 0.01, *** *p* < 0.001 vs. I/R group.

Bielschowsky silver staining (BSSM) was employed to evaluate axonal pathology due to the argyrophilic properties of neuronal fibers. Axons appeared dark brown, allowing visualization of the filamentous nerve fiber network. In the I/R group, numerous axonal retraction bulbs (red arrows), axonal swelling (yellow arrows), and nerve fiber retraction were observed (Figure 5A). These pathological alterations at synaptic sites were markedly attenuated in the CG- and FH-treated groups. Semi-quantitative scoring indicated significantly reduced axonal loss in CG-treated rats (Figure 5C), with no significant difference observed between the CG and FH groups, demonstrating comparable protective effects.

### 2.5. CG Facilitates Axonal Regeneration After I/R Injury

Immunofluorescence (IF) was employed to evaluate the expression of axonal structural proteins, including NF-200, MAP2, and GAP43, as indicators of axonal regeneration. As shown in Figure 5B, strong protein expression was observed in the Sham group, whereas it was markedly reduced in the I/R model group, indicating severe axonal damage following ischemia/reperfusion injury. Seven days of CG administration significantly increased the expression of these proteins in a dose-dependent manner (Figure 5B, *** *p* < 0.001). Together, the findings from both BSSM and IF analyses indicate that CG promotes axonal regeneration and structural remodeling during recovery from I/R injury.

### 2.6. CG Inhibited Glial Scar Formation and Reduced CSPG Expression After Ischemic Stroke

**Figure 6:**
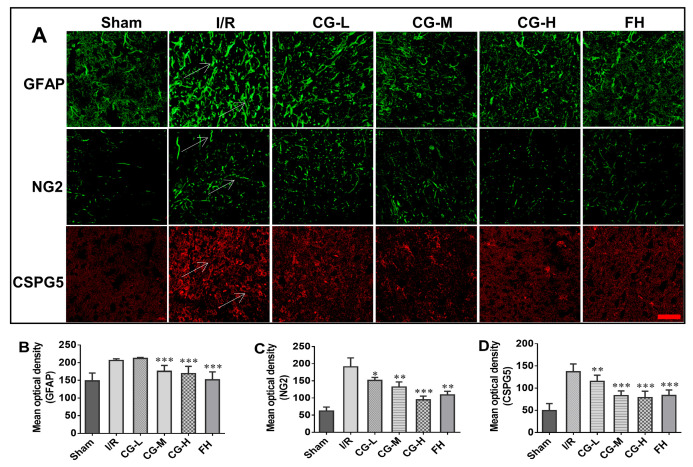
IF staining in tissues from rats sacrificed on day 7 after I/R injury. (**A**) Representative GFAP, NG2 and CSPG5 fluorescence staining. Scale bar = 40 μm. (**B**–**D**) Semi-quantitative analysis of fluorescence intensity of GFAP, NG2, and CSPG5. Data are presented as mean ± SD (n = 5). * *p* < 0.05, ** *p* < 0.01, *** *p* < 0.001 vs. I/R group.

GFAP, a key component of the glial scar, was used to assess astrocyte activation and glial scar formation in the peri-infarct cortex and hippocampus. Positive staining was quantified in predefined anatomical regions using representative fields selected under the same criteria for all groups, and representative positive signals are indicated by white arrows in Figure 6A. As shown in Figure 6A,B, GFAP expression was significantly reduced in the CG-M and CG-H groups compared with the I/R group, indicating that middle- and high-dose CG suppressed astrocyte activation and glial scar formation after ischemic stroke.

CSPGs, including CSPG4 and CSPG5, are typically upregulated after cerebral ischemia and contribute to an extracellular environment that restricts axonal extension. Rat NG2 is a homolog of human CSPG4. Quantitative analysis of staining intensity in the same anatomical regions showed that CG treatment markedly reduced CSPG expression compared with the I/R group, and representative positive signals are indicated by white arrows in Figure 6A. These results suggest that CG reduced the expression of extracellular inhibitory molecules associated with axonal growth restriction.

### 2.7. CG Promotes Axonal Regeneration via Rho/ROCK Signaling Pathway After I/R Injury

**Figure 7:**
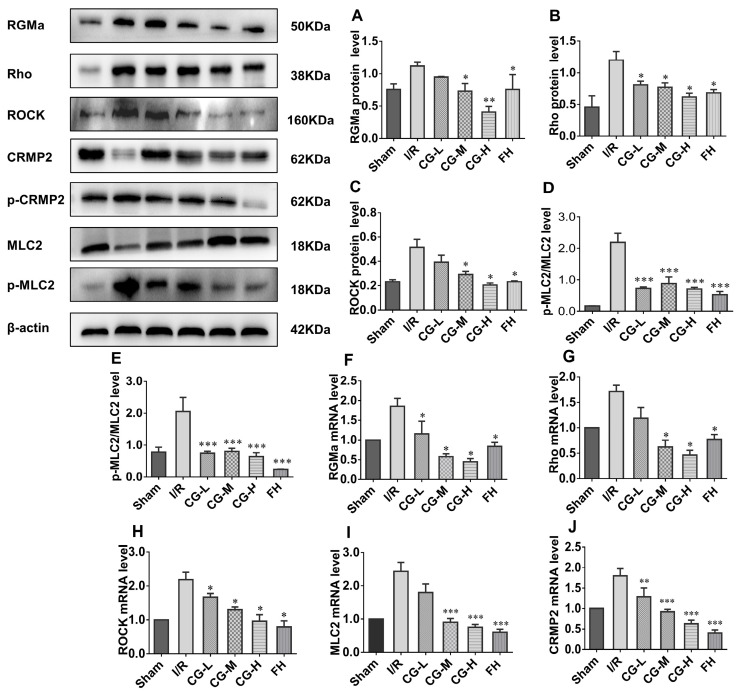
CG dependence on the Rho/ROCK signaling pathway after MCAO injury. (**A**–**D**) Protein expression of RGMa, Rho, ROCK, and MLC2; n = 3. (**E**–**J**) mRNA levels of MLC2, RGMa, Rho, ROCK, MLC2 and CRMP2; n = 5. Data are presented as mean ± SD. * *p* < 0.05, ** *p* < 0.01, *** *p* < 0.001 vs. I/R group.

Our results demonstrate that CG facilitates recovery from neurological deficits following ischemic stroke, with axonal regeneration playing a critical role in this process. To investigate whether the Rho/ROCK signaling pathway is involved, Western blot (WB) and qRT-PCR analyses were performed to assess the expression of RGMa, Rho, ROCK, MLC2, and CRMP2. Semi-quantitative analysis of WB data (Figure 7A–E) revealed that protein levels of RGMa, Rho, and ROCK, as well as the phosphorylated forms of MLC2 and CRMP2, were significantly reduced in CG- and FH-treated groups compared with the I/R group. Consistently, mRNA levels of RGMa, Rho, ROCK, MLC2, and CRMP2 were markedly decreased in the CG and FH groups relative to I/R controls (Figure 7F–J). Collectively, these findings indicate that CG promotes axonal regeneration, at least in part, by inhibiting the Rho/ROCK signaling pathway following ischemic stroke.

## 3. Discussion

In this study, we elucidated the mechanisms by which CG reduces cerebral infarct volume and promotes neurological recovery in rats with ischemic stroke (IS), highlighting a critical role for axonal regeneration. Necrosis in the central infarct zone is irreversible even shortly after ischemia [14]; however, the surrounding ischemic penumbra (IP) remains electrically inactive yet preserves normal transmembrane potentials. Timely restoration of cerebral blood flow can salvage the IP, thereby limiting infarct expansion [15]. Therefore, the reduction in infarct volume observed after CG treatment may suggest a protective effect on the ischemic penumbra.

Cerebral ischemia leads to extensive neuronal loss, with estimates indicating that 1 h of ischemia can damage approximately 120 million neurons, 830 billion synapses, and 714 km of myelinated fibers [16]. In the early post-infarction period, synaptic structures in the IP and distal functional regions are also compromised due to tissue edema, reduced perfusion, and metabolic disturbances [17]. Consistently, I/R rats exhibited axonal retraction bulbs and reduced neuronal density, indicative of axonal damage and resulting neurological deficits. Acute IS also impairs neural stem cell proliferation in the subventricular zone and striatum [18,19]; although these cells are capable of renewal, ischemia limits their maturation and synaptic integration due to energy and nutrient deficits [20]. Therefore, functional recovery relies heavily on the sprouting and extension of intact axons in peri-infarct regions to compensate for lost fiber tracts [21,22,23]. In our study, CG reduced axonal retraction and swelling, facilitated axonal extension, and restored neurological function, suggesting that CG promotes axonal sprouting to re-establish neuronal connectivity.

The axonal growth cone, composed of microtubules, microfilaments, and myosin, is highly sensitive to extracellular cues [24]. Cytoskeletal proteins are degraded following ischemic injury. NF-200, an abundant intermediate filament in axons, serves as a marker of axonal elongation and reflects neuronal morphology after injury [25,26]. Upregulation of NF-200 by CG indicates enhanced axonal extension. MAP2, a microtubule-associated protein distributed in neuronal somas, dendrites, and axons, is critical for microtubule polymerization and stability, promoting assembly, repair, and regeneration of damaged axons [27]. Increased MAP2 expression in CG-treated rats suggests augmented axonal formation. GAP43, localized to axonal growth cones during development and regeneration, is rapidly upregulated following neuronal injury and trafficked from the soma to axons until synaptic connections are re-established [28]. Our data confirmed that CG upregulated NF-200, MAP2, and GAP43, supporting its role in stabilizing the neuronal cytoskeleton and promoting axonal regeneration.

The Rho/ROCK pathway is a central regulator of cytoskeletal dynamics and axonal regeneration [29]. RGMa functions upstream of this pathway, and its inhibitory effects on growth cones can be reversed by Rho or ROCK inhibitors [30]. In spinal cord injury models, RGMa neutralization promotes axonal growth and motor recovery. CG downregulated RGMa expression, suggesting potential RGMa inhibitory activity. CRMP2, localized in neuronal somas, axons, and dendrites, acts downstream of Rho/ROCK to regulate microtubule assembly and dynamics during axonal growth [31,32]. ROCK activation after ischemia inhibits CRMP2-tubulin binding, impairing axonal elongation and contributing to neuronal death [33]. In our study, CG decreased RGMa and Rho expression while upregulating CRMP2 in MCAO rats. Collectively, these findings suggest that CG facilitates axonal regeneration and neurological recovery, at least in part, by inhibiting the Rho/ROCK signaling pathway.

Although the morphological and cellular alterations observed in this study may be indicative of axonal remodeling, they cannot be considered conclusive evidence of axonal growth per se. The observed attenuation of axonal injury and the increased expression of axonal regeneration-related markers suggest a pro-regenerative effect of CG; however, further studies are required to determine whether these changes are truly associated with axonal elongation, target reconnection, and functional integration. Future investigations using more direct assessments, such as axonal tracing, high-resolution imaging, electrophysiological analysis, and longer-term observation, will be important to further validate the relationship between these changes and true axonal growth after ischemic stroke.

A notable feature of the present study is the use of a 7-day observation period following ischemic stroke. This time frame was chosen in consideration of the pathophysiological characteristics of acute ischemic injury, as the first 7 days after stroke are widely recognized as a critical period for penumbral salvage, early axonal repair, and initial neurological recovery. Moreover, this interval closely parallels the acute intervention window in clinical practice. Within this period, our results clearly demonstrated that CG rapidly reduced infarct volume, mitigated axonal injury, and upregulated molecular signals associated with axonal regeneration, supporting its acute neuroprotective and pro-regenerative effects in the early stage after ischemic stroke. Importantly, the present findings still have considerable translational relevance. The 7-day observation window corresponds to the clinically important early phase of stroke treatment, during which rapid intervention is essential to preserve viable tissue and initiate repair processes. In this context, the ability of CG to reduce acute brain injury and promote axonal regeneration suggests that it may have potential as an early therapeutic strategy for ischemic stroke. Although the present findings support the therapeutic potential of CG in acute ischemic stroke, its translational application will also depend on a clearer understanding of its feasibility and safety profile. As a purified bioactive constituent of Astragalus Radix, CG may be amenable to standardization and further pharmacological development. However, the current study did not address toxicological safety, pharmacokinetics, or dose-limiting effects. These issues should be systematically investigated in future studies to better define the clinical potential of CG. In addition, the mechanistic evidence supporting CG-mediated activation of axonal repair pathways provides a rationale for its further evaluation in acute-phase translational and clinical studies. The early improvement in neurological function observed in this study may also be clinically meaningful, as effective intervention during the acute stage could help reduce early neurological deficits and create favorable conditions for subsequent long-term recovery.

## 4. Materials and Methods

### 4.1. Reagents

Calycosin-7-O-β-D-glucoside (CG, Cat# CHB180110) was provided by Chengdu Chroma-Biotechnology Co., Ltd. (Chengdu, China). Fasudil hydrochloride (FH) was obtained from Neuraxpharm (Arzneimittel GmbH, Langenfeld, North Rhine-Westphalia, Germany). Triphenyltetrazolium chloride (TTC) was purchased from Shanghai Yuanye Bio-Technology Co., Ltd. (Shanghai, China). The Hematoxylin and Eosin (HE) staining kit, Nissl staining kit, bicinchoninic acid (BCA) protein assay kit, radioimmunoprecipitation assay (RIPA) buffer, and antifade mounting medium were supplied by Beyotime Biotechnology (Shanghai, China). Optimal cutting temperature (OCT) compound was obtained from Sakura Finetek USA Inc. (Torrance, CA, USA).

Primary antibodies against GAP43 (Cat# ab75810), RGMa (Cat# ab307652), and Rho (Cat# ab98887) were purchased from Abcam (Cambridge, UK); antibodies against NF-200 (Cat# 30564), MAP2 (Cat# 4542) and MLC2 (Cat# 3672) were obtained from Cell Signaling Technology (CST, Boston, MA, USA); antibodies against GFAP (Cat# DF6040), CSPG4 (Cat# DF12589), CSPG5 (Cat# DF3938), ROCK (Cat# AF7016), phosphorylated MLC2 (p-MLC2, Cat# AF8618), CRMP2 (Cat# AF6459), phosphorylated CRMP2 (p-CRMP2, Cat# AF3459), and β-actin (Cat# AF7018) were supplied by Affinity Biosciences (Cincinnati, OH, USA). Alexa Fluor 594 (Cat# S0006) and FITC-conjugated secondary antibodies (Cat# S0008) were also obtained from Affinity Biosciences (Cincinnati, OH, USA). DAPI (Cat# C1006-10 mL) was obtained from Beyotime Biotech. Inc. (Beijing, China). The Glodenstar™ RT6 cDNA Synthesis Mix Kit and 2× T5 Fast qPCR Mix (SYBR Green I) were purchased from Tsingke Bio-Technology Co., Ltd. (Guangzhou, China).

### 4.2. Animals and Induction of I/R Injury

Male Sprague–Dawley rats (250–280 g; license number: SCXK (Guangdong) 2013-0034) were obtained from the Experimental Animal Center of Guangzhou University of Chinese Medicine (Guangzhou, China). Animals were housed under controlled environmental conditions (constant temperature and humidity) with a 12 h light/dark cycle and allowed to acclimatize for 7 days prior to experimentation. All experimental procedures were conducted in accordance with the National Institutes of Health Guide for the Care and Use of Laboratory Animals (NIH Publication No. 8023, revised 1978).

Prior to surgery, rats were fasted for 12 h with free access to water. Transient focal cerebral ischemia was induced using a modified intraluminal filament middle cerebral artery occlusion (MCAO) model as previously described by Longa et al. [34]. Briefly, following anesthesia, animals were placed in the supine position on a heated operating table, and body temperature was maintained at 37 °C using an electric heating blanket. The left common carotid artery (CCA), external carotid artery (ECA), and internal carotid artery (ICA) were carefully exposed. The CCA and ECA were ligated to prevent bleeding. A small incision was made in the distal CCA, through which a 2.66 mm nylon monofilament suture with a polylysine-coated tip was gently advanced into the ICA via the CCA. The filament was inserted to a depth of 18–20 mm from the carotid bifurcation, thereby occluding the origin of the middle cerebral artery and inducing focal ischemia for 2 h. After surgery, penicillin was administered to prevent infection.

Sham-operated rats underwent the same surgical procedures, including vessel exposure and ligation, but without insertion of the nylon filament.

### 4.3. Experimental Groups and Drug Administration

Neurological function was evaluated after recovery from anesthesia using a modified neurological deficit scoring system based on the method described by Bederson et al. [35], and animals with unsuccessful modeling were excluded. Rats with neurological scores ranging from 1 to 3 were selected and randomly allocated to ensure comparable baseline neurological deficits among groups.

Eligible animals were randomly assigned to six groups (n = 20 per group) for intraperitoneal administration: Sham control, ischemia/reperfusion (I/R), CG low-dose (CG-L, 15 mg/kg), CG middle-dose (CG-M, 30 mg/kg), CG high-dose (CG-H, 60 mg/kg), and fasudil hydrochloride (FH; positive control, 10 mg/kg). CG and FH were dissolved in dimethyl sulfoxide (DMSO) and further diluted with sterile normal saline containing 0.5% Tween-20. Animals received daily intraperitoneal injections of the corresponding drugs or vehicle (Sham and I/R groups) for 7 consecutive days.

### 4.4. Neurological Function Scoring

Behavioral assessments were conducted on days 1, 3, 5, and 7 after the MCAO procedure to evaluate the effects of CG on neurological function. Neurological deficits were quantified using the modified neurological severity score (mNSS) based on the method described by Garcia et al. [36]. The mNSS evaluation included assessments of spontaneous activity, limb movement, forepaw extension, climbing ability, tactile response, and vibrissae stimulation. The final mNSS score was calculated as the sum of these six components, with a maximum score of 18 and a minimum score of 3, where lower scores indicate more severe neurological impairment.

Motor balance and coordination were further evaluated using the balance beam test (BBT). Briefly, rats were required to traverse a narrow wooden beam (80 cm in length and 1.5 cm in width) suspended 15 cm above a cushioned platform. Prior to model induction, animals underwent daily training sessions until they were able to traverse the beam smoothly. Neurological impairment was scored using a standardized 7-point grading system [37]: 0, maintained balance with both limbs without falling; 1, grasped the beam with the forelimbs and remained on the beam; 2, rolled to one side but was still able to remain on the beam; 3, rolled to both sides and remained on the beam for <60 s; 4, rolled to both sides and remained on the beam for <40 s; 5, rolled to both sides and remained on the beam for <20 s; and 6, fell directly from the beam. Higher scores indicate more severe impairment in motor balance and coordination.

### 4.5. Infarct Volume Measurement

Rats were deeply anesthetized and euthanized, after which the brains were rapidly removed and immediately frozen at −20 °C for 30 min. Each brain was coronally sectioned into six slices, which were subsequently incubated in 2% triphenyltetrazolium chloride (TTC) solution in the dark at 37 °C for 30 min. Following TTC staining, the brain slices were rinsed with phosphate-buffered saline (PBS, pH 7.4). The posterior surface of each slice was imaged, and infarct areas were quantified using ImageJ software version 1.54s (Rawak Software Inc., Stuttgart, Baden-Wurttemberg, Germany).

Total infarct volume and infarct rate were calculated as previously described [38]. The infarct rate was determined using the following formula: (volume of non-infarcted hemisphere − volume of non-infarcted volume in infarcted hemisphere)/volume of non-infarcted hemisphere × 100%.

### 4.6. Brain Water Content

After the final drug administration, rats were sacrificed by decapitation, and the whole brains were rapidly removed and sectioned into 3-mm-thick coronal slices. The fresh brain tissues were immediately weighed using an electronic analytical balance to obtain the wet weight. Subsequently, the slices were placed on pre-baked aluminum foil and dried in an oven at 102 °C for 12 h to obtain the dry weight.

Brain water content was calculated using the following formula: (wet weight − dry weight)/wet weight × 100%.

### 4.7. Pathological Assessment

After 7 days of drug administration, histopathological evaluation of brain tissue and neuronal injury was performed using hematoxylin and eosin (HE) staining and Nissl staining. Briefly, rats were deeply anesthetized and transcardially perfused with cold saline, followed by fixation with 4% paraformaldehyde. Brains were then rapidly removed and immediately frozen. The frozen tissues were post-fixed and sectioned into 10-μm-thick coronal slices using a freezing microtome (Thermo Fisher Scientific, Waltham, MA, USA).

The sections were rinsed with distilled water and stained with an HE staining kit or Nissl staining solution according to the manufacturers’ protocols. Histological images of the cortex and hippocampus in the infarcted hemisphere were captured using a light microscope (Olympus Corporation, Tokyo, Japan). Neuronal morphology and survival were quantitatively analyzed using ImageJ software [39], with neurons identified by the presence of a clearly defined nucleolus and abundant Nissl bodies.

### 4.8. Bielschowsky’s Silver Staining (BSSM)

Axonal degeneration was assessed using the Bielschowsky silver staining method (BSSM). Frozen sections were sequentially incubated in silver nitrate (AgNO_3_) solution and Glee’s solution, followed by conventional dehydration, clearing, and mounting procedures, according to a modified protocol described by Segura-Anaya et al. [40].

Axonal loss was evaluated using a semi-quantitative scoring system as previously reported by Zeinali et al. [41], defined as follows:

0 = no axonal loss;
1 = mild superficial axonal loss involving <25% of the tissue;
2 = moderate deep axonal loss involving >25% of the tissue;
3 = diffuse and extensive axonal loss involving >50% of the tissue.

### 4.9. Immunofluorescence (IF)

Immunofluorescence staining of NF-200, MAP2, and GAP43 was performed to evaluate neurite growth, while GFAP and chondroitin sulfate proteoglycans (CSPGs) were used to assess axonal inhibitory signaling. Frozen brain sections were rinsed with phosphate-buffered saline (PBS) and permeabilized with 0.3% Triton X-100. The sections were then washed with PBS, blocked with 10% goat serum, and incubated overnight at 4 °C with the following primary antibodies: NF-200 (1:50), MAP2 (1:250), GAP43 (1:500), GFAP (1:100), CSPG4 (1:100), and CSPG5 (1:100).

After primary antibody incubation, sections were washed with PBS containing 0.1% Tween-20 (PBST) and incubated with the appropriate fluorescent secondary antibodies—Alexa Fluor 594 (1:200) or FITC (1:250). Nuclei were counterstained with DAPI (5 μg/mL). The sections were then mounted with antifade mounting medium and visualized using a laser scanning confocal microscope (Carl Zeiss AG, Oberkochen, Germany).

### 4.10. Western Blot (WB)

Total proteins were extracted from brain tissue samples using radioimmunoprecipitation assay (RIPA) buffer. Lysates were centrifuged, and protein concentrations in the supernatants were determined using a bicinchoninic acid (BCA) protein assay kit. Equal amounts of protein were separated by sodium dodecyl sulfate–polyacrylamide gel electrophoresis (SDS–PAGE) and subsequently transferred onto polyvinylidene fluoride (PVDF) membranes.

The membranes were blocked with Tris-buffered saline containing 0.1% Tween-20 (TBST) supplemented with 5% skim milk and incubated overnight at 4 °C with the following primary antibodies: RGMa (1:15,000), Rho (1:2500), and CRMP2 (1:5000). β-actin (1:1000) was used as the internal loading control. After washing, membranes were incubated with horseradish peroxidase (HRP)-conjugated goat anti-rabbit IgG secondary antibody (1:5000). Protein bands were visualized using an enhanced chemiluminescence (ECL) detection system and imaged with a Tanon imaging system (Shanghai Tanon Life Science Co., Ltd., Shanghai, China). Band densities were quantified using ImageJ software. The protein expression levels were normalized to the expression level of β-actin. For phosphorylated proteins, the expression levels are additionally presented as the ratio of phosphorylated protein to total protein where appropriate.

### 4.11. Quantitative Real-Time Polymerase Chain Reaction (qRT-PCR)

After 7 days of CG administration, total RNA was extracted from brain tissues using TRIzol reagent (Yeasen Biotechnology Co., Ltd., Shanghai, China), and complementary DNA (cDNA) was synthesized with the Glodenstar™ RT6 cDNA Synthesis Mix Kit according to the manufacturer’s instructions. Quantitative real-time PCR (qRT-PCR) was performed using the 2× T5 Fast qPCR Mix (SYBR Green I) Kit. Gene expression levels were quantified using the comparative Ct method (2−ΔΔCt), with β-actin serving as the internal reference. Primer sequences used for qRT-PCR are listed in Table 1.

### 4.12. Data Analysis

All statistical analyses were performed using SPSS 22.0 (IBM, Armonk, NY, USA) and GraphPad Prism 7.0 (GraphPad Software, San Diego, CA, USA). Comparisons among multiple groups were conducted using one-way analysis of variance (ANOVA), followed by post hoc tests where appropriate, while differences between two groups were assessed using the unpaired Student’s *t*-test. Data are presented as mean ± standard deviation (SD), and differences were considered statistically significant at *p* < 0.05. Statistical annotations in the figures are defined as follows: *p* < 0.05 (*), *p* < 0.01 (**), and *p* < 0.001 (***).

## 5. Conclusions

In summary, our study demonstrates that CG promotes axonal regeneration and facilitates neurological recovery following cerebral ischemia. CG-treated rats exhibited significant improvements in behavioral performance, reduced nerve fiber disruption, and attenuated axonal degeneration. Furthermore, the expression of NF-200, MAP2, and GAP43 was markedly upregulated. These neuroprotective and regenerative effects are mediated, at least in part, through inhibition of the Rho/ROCK signaling pathway. Collectively, our findings not only advance the mechanistic understanding of CG in neural regeneration but also provide compelling evidence supporting its potential as a therapeutic intervention for ischemic stroke.

## Figures and Tables

**Table 1:** Sequences of primers.

	Primer Sequences (5′-3′)	Length of Product
β-actin	F: AGGGAAATCGTGCGTGACAT R: GAACCGCTCATTGCCGATAG	150 bp
RGMa	F: TCCAGACATGTAAGGTGCAA R: ACTTTCTGGTCCACACACTCT	160 bp
Rho	F: TATTGAAGTGGACGGGAAGCA R: AACTATCAGGGCTGTCGATGGA	140 bp
ROCK	F: GCTCAAGACATGCTCAATCA R: ACATGGCAACAGACTTTGC	178 bp
CRMP2	F: ACCAACGCAGCCAAAGTCTT R: GAGCACTGTTGTGCGTCTTG	130 bp

## Data Availability

Data is contained within the article and Supplementary Material.

## Supplementary Materials

The following supporting information can be downloaded at https://www.mdpi.com/article/10.3390/ijms27104469/s1.
