# Supplementary material for: Calycosin-7-O-β-D-Glucoside Facilitates Axonal Regrowth and Functional Recovery via Rho/ROCK Pathway Inhibition After Cerebral Ischemia/Reperfusion
Source: Int J Mol Sci. 2026 May 16;27(10):4469. doi: 10.3390/ijms27104469 (PMC13207909; doi:10.3390/ijms27104469)
Supplement: Supplementary file 1 [file ijms-27-04469-s001.zip › ijms-4244989-supplementary.pdf]

Raw data

Figure7A

RGMa1

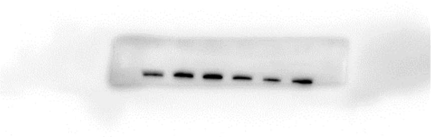

RGMa2

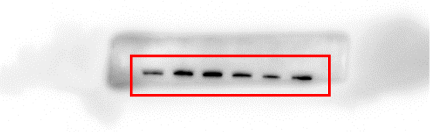

RGMa3

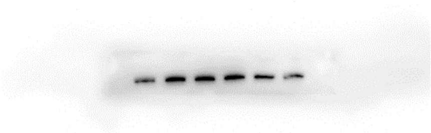

RGMa4

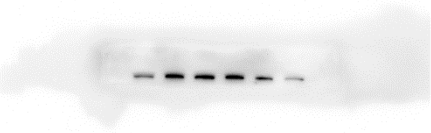

Actin

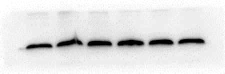

Figure7A

Rho1

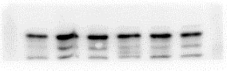

Rho2

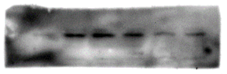

Rho3

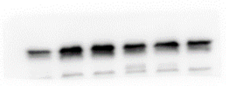

Rho4

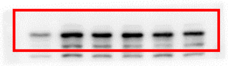

Actin

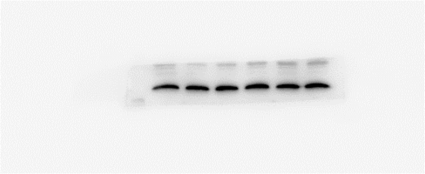

Figure7A

ROCK1

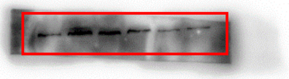

ROCK2

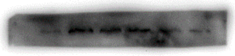

ROCK3

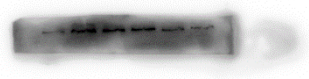

ROCK4

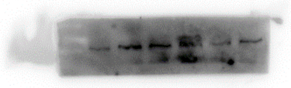

Actin

Figure7A

CRMP2（1）

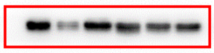

CRMP2（2）

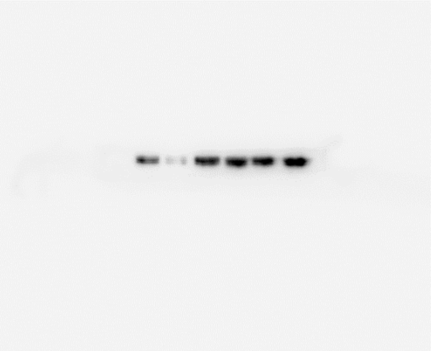

CRMP2（3）

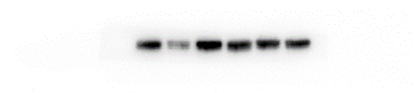

Actin

Figure7A

p-CRMP2（1）

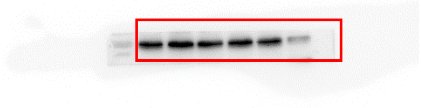

p-CRMP2（2）

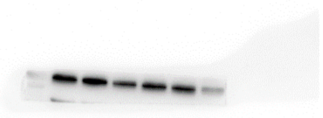

p-CRMP2（3）

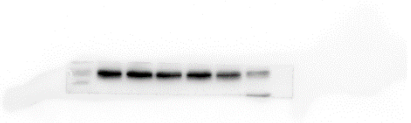

MLC1

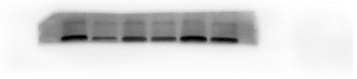

MLC2

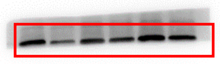

MLC3

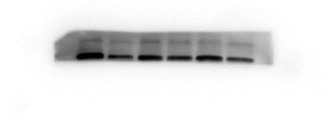

MLC4

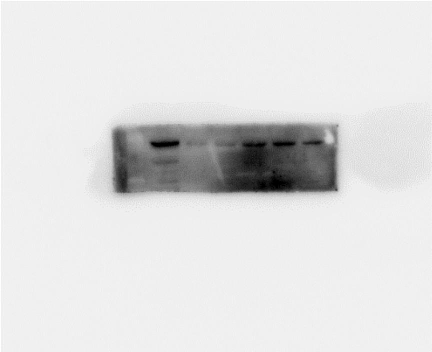

Actin

p-MLC1

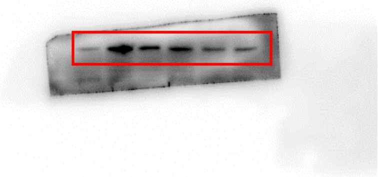

p-MLC2

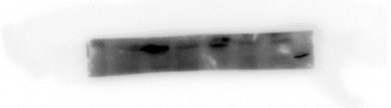

p-MLC3

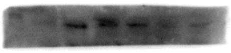

Actin
